# Evaluation of Serum Leptin Levels and Growth in Patients with *β*-Thalassaemia Major

**DOI:** 10.1155/2016/8454286

**Published:** 2016-03-21

**Authors:** Lamia Mustafa Al-Naama, Meaad Kadum Hassan, Muhannad Maki Abdul Karim

**Affiliations:** ^1^Department of Biochemistry and Haemoglobinopathy Units, College of Medicine, University of Basrah, Basrah, Iraq; ^2^Department of Paediatrics and Haemoglobinopathy Units, College of Medicine, University of Basrah, Basrah, Iraq; ^3^Department of Biochemistry, College of Medicine, University of Basrah, Basrah, Iraq

## Abstract

*Background*. Iron deposition in the body can damage the endocrine glands of patients with *β*-thalassaemia major (*β*-TM). Leptin plays a key role in the regulation of appetite, body fat mass, and endocrine function.* Objectives*. This study aimed to evaluate the relationship between serum leptin and growth and pubertal development in patients with *β*-TM, as well as whether serum leptin can predict growth retardation and delayed puberty in these patients.* Methods*. Fifty *β*-TM patients (aged 8–20 years) and 75 age-matched healthy controls were recruited. Anthropometric data and sexual maturity ratings were assessed. Serum leptin was measured by ELISA.* Results*. Serum leptin levels were significantly lower in patients with *β*-TM than in healthy individuals (*P* < 0.001). Leptin levels were also significantly reduced in female patients with short stature (*P* < 0.002) and in patients who displayed delayed puberty (*P* = 0.032) compared to those with normal stature who had reached puberty. The sensitivity of leptin for predicting short stature and delayed puberty among patients was 84.6% and 92.3%, respectively.* Conclusion*. Low serum leptin is sensitive to predict short stature and significant in *β*-TM females only. This link could thus be used as a guide for further therapeutic or hormonal modulation.

## 1. Introduction

Short stature among patients with thalassaemia is a problem in developing countries due to several factors, such as inadequate blood transfusion, iron overload, abnormal growth hormone (GH) secretion, hypothyroidism, zinc deficiency, deferoxamine toxicity, inadequate treatment, and noncompliance of patients [1].

Leptin, an adipokine that is synthesized and released from adipocytes in response to changes in body fat [2], binds receptors within the hypothalamus to control appetite [2–4]. Moreover, leptin stimulates the secretion of luteinising hormone (LH) by activating nitric oxide synthase in gonadotropes and in the hypothalamus, which in turn stimulates the release of gonadotropin-releasing hormone (GnRH) [5, 6]. Leptin has been proposed as a physiological link between nutritional status and reproductive maturation and function [2]. Therefore, leptin may serve as a trigger or metabolic gate for sexual development [7–9]. The importance of leptin in regulating sexual maturation is supported by data showing that alterations in the leptin receptor or deletion of the leptin gene results in infertility [10], whereas the administration of leptin to leptin-deficient patients reportedly led to increased serum gonadotropin hormone levels [11].

Previous studies have demonstrated that leptin can be considered one of the many metabolic signals that can regulate growth hormone (GH) secretion [12]. In rats, the central administration of leptin completely prevents the disappearance of pulsatile GH secretion that occurs after 3 days of fasting [12].

Beta-thalassaemia (*β*-TM) is a hereditary disorder that is highly prevalent worldwide and in our local community of Basrah, Iraq [13], due to high rates of relative marriages. Therefore, we conducted this study to evaluate whether serum leptin levels in Iraqi patients with *β*-TM from Basrah correlate with short stature, body mass index (BMI), and delayed puberty relative to normal children as well as study the independent effects of selected risk factors on serum leptin levels. Furthermore, we examined the sensitivity of serum leptin levels in predicting growth retardation and delayed puberty in patients with *β*-TM.

## 2. Patients and Methods

### 2.1. Patients

This case-control study was conducted between December 1, 2008, and March 31, 2010. The Council and the Ethical Committee of the College of Medicine at the University of Basrah approved the protocol for this study. The study was explained to the young adult patients or controls and their parents/relatives or guardian, and appropriate written consent was obtained.

The patient group comprised 50 patients (27 males and 23 females) diagnosed with *β*-TM using HPLC Variant (Bio-Rad, USA) who were registered at the Center for Hereditary Blood Diseases in Basrah, Iraq. Patients undergoing hormonal or zinc therapy were excluded.

The control group included 75 age-matched healthy individuals (41 males and 34 females) with normal haemoglobin (Hb AA) and no previous history of relevant medical illnesses, such as a history of anaemia.

### 2.2. Anthropometry

Body weight, height, body mass index (BMI), and BMI *Z*-score (BMIZ) were assessed and plotted on age- and sex-appropriate growth charts. Short stature was defined according to the Centers for Disease Control and Prevention (CDC) as height or stature below the 5th percentile on the CDC age- and gender-specific height or stature reference [14]. BMI and BMI *Z*-score were calculated for all patients according to the WHO 2006/2007 reference values [15]. BMI *Z*-scores were used to determine the severity of wasting. Pubertal staging was assessed using the sex maturity rating (SMR) according to Tanner classification [16], wherein any person with signs of stage 2 or greater, plus breast development in girls or genital development in boys, was allocated to pubertal groups. An individual was considered to have delayed puberty when a girl of 13 or a boy of 14 did not exhibit signs of pubertal development, that is, the absence of breast development in a girl by the age of 13 or a testicular volume of less than 4 mL in a boy by the age of 14. Other definitions of delayed puberty included the absence of menarche by 16 years of age or a prolonged rate of pubertal progression with more than 5 years from pubertal onset to completion [16].

### 2.3. Laboratory Analysis

Blood samples were collected after overnight fast from 8:30 to 9:30 a.m. to ensure uniform timing for sample collection. The blood was centrifuged, and the sera were stored at −18°C until assayed.

Serum leptin was measured in duplicate by enzyme-linked immunosorbent assay (ELISA) using a kit from DRG (Germany). The inter- and intra-assay coefficients of variation were 5.3% and 4.9%, respectively. Based on the values of the control individuals, the reference values for males and females were 3.92 ± 1.22 ng/mL and 5.95 ± 2.43 ng/mL, respectively. A low serum leptin level is defined as a value below the 95% confidence intervals of the mean values of the control group according to the sex. Serum ferritin levels were estimated by ELISA using a kit from HUMAN (Germany).

### 2.4. Statistical Analysis

Data were analysed using Statistical Package for the Social Sciences (SPSS) software version 15 (IBM, Chicago, Illinois, USA), and the results are presented as tables or figures. For continuous variables, means and standard deviations were used to present the data, and analysis was performed using independent Student's *t*-test. The strength and direction of linear relationships between variables were evaluated using Pearson's correlation coefficient. A linear multiple regression analysis was used to identify significant predictors of changes in serum leptin. The validity of serum leptin estimation as a predicator of short stature and delayed puberty among *β*-TM patients was assessed using receiver operating characteristic (ROC) analyses, and the area under the curve (AUC) was calculated. *P* < 0.05 was considered statistically significant.

## 3. Results

The mean serum leptin level, BMI, BMI *Z*-score, height, and weight were significantly lower in patients of both sexes with *β*-TM than in controls (*P* < 0.014 to *P* < 0.001) (Table 1). The mean serum leptin level was significantly higher in female *β*-TM patients than in male *β*-TM patients (*P* < 0.001). However, within the patient group, there were no significant differences in BMI, BMI *Z*-score, and weight between males and females. As expected, serum ferritin levels were significantly higher (*P* = 0.001) in *β*-TM patients relative to healthy control individuals, with no significant difference (*P* > 0.05) between male and female patients (Table 1).

Concerning delayed puberty, 7 of 11 (63%) male patients at least 14 years of age and 6 of 11 (54.5%) female patients at least 13 years of age showed signs of delayed puberty. In contrast, all individuals in the control group exhibited normal pubertal development. All male *β*-TM patients with delayed puberty (100%) and 5 of 6 females with delayed puberty (83.3%) had low serum leptin levels. The mean serum leptin level was significantly lower in all patients with delayed puberty (*P* = 0.032) (Table 2).

Short stature was observed in 26 of 50 (52%) patients with *β*-TM. Low serum leptin levels were detected in 33 of 50 (66%) *β*-TM patients, of whom 22 (67%) had short stature, whereas the rest had normal stature. The mean serum leptin level was significantly lower only in female patients with short stature (*P* = 0.002) (Table 3).

**Figure 1:**
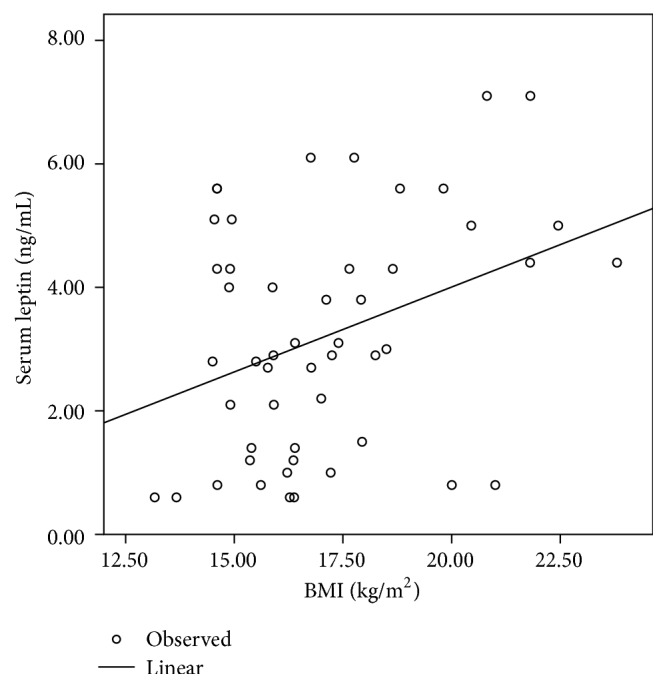
Correlation between serum leptin levels (ng/mL) and BMI (kg/m^2^) in *β*-TM patients. Pearson correlation coefficient (*r* = 0.353, *P* = 0.012).

**Figure 2:**
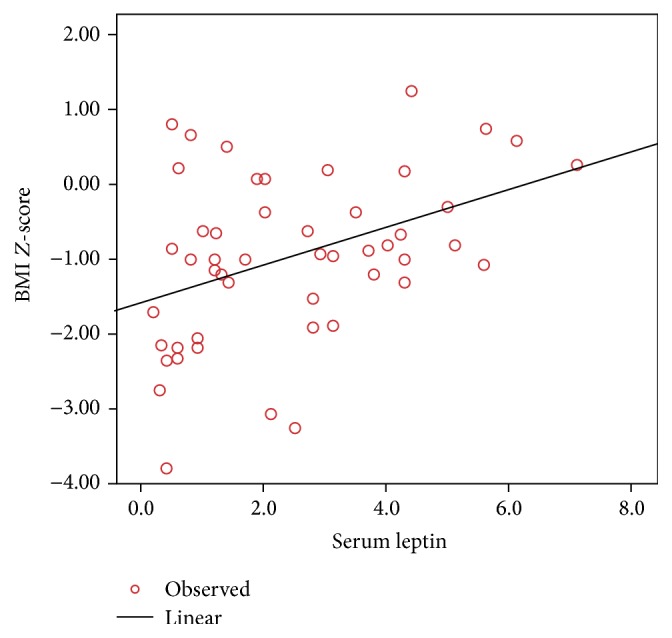
Correlation between serum leptin levels (ng/mL) and the BMI *Z*-score in *β*-TM patients. Pearson correlation coefficient (*r* = 0.404, *P* = 0.004).

In *β*-TM patients, a significant positive correlation was observed between serum leptin levels and BMI (*r* = 0.353, *P* = 0.012) (Figure 1), as well as with the BMI *Z*-score (*r* = 0.404, *P* = 0.004) (Figure 2).

To study the independent effects of selected risk factors on serum leptin levels, a stepwise linear multiple regression analysis was performed (Table 4). The factors examined were age, sex, *β*-TM status, BMI *Z*-score, and BMI. *β*-TM, sex, and BMI were significantly correlated with serum leptin levels, with *β*-TM appearing to be the strongest variable, accounting for 25.1% of the variability in serum leptin levels. Combined variables (*β*-TM, sex, and BMI) explained 45.6% of the variation in serum leptin levels, whereas 54.4% of the variation remained unaccounted for.

**Figure 3:**
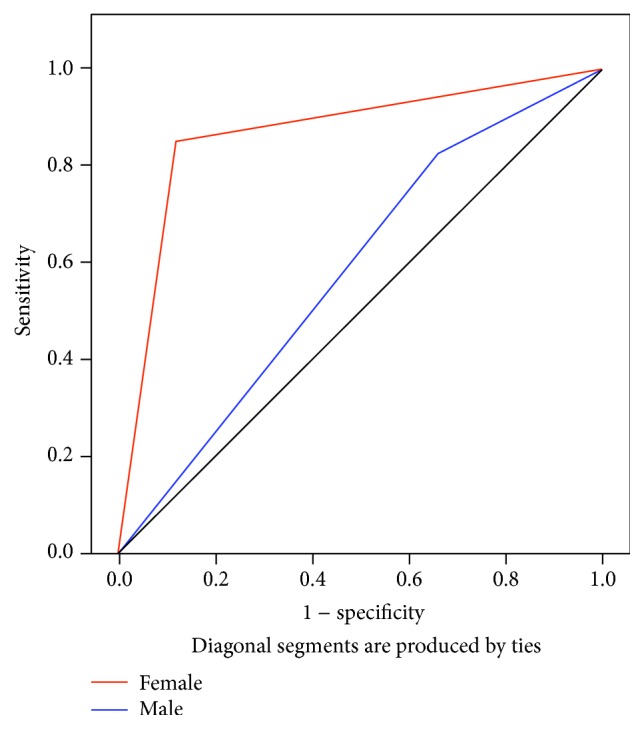
Receiver operating characteristic (ROC) curves of serum leptin levels and short stature in *β*-TM patients using cut-off values mentioned in the text. The red and blue lines indicate female and male *β*-TM patients, respectively. The AUCs (±SE) and *P* values for female and male *β*-TM patients are 0.822 (±0.095), *P* = 0.009 and 0.638 (±0.116), *P* = 0.265, respectively.

**Figure 4:**
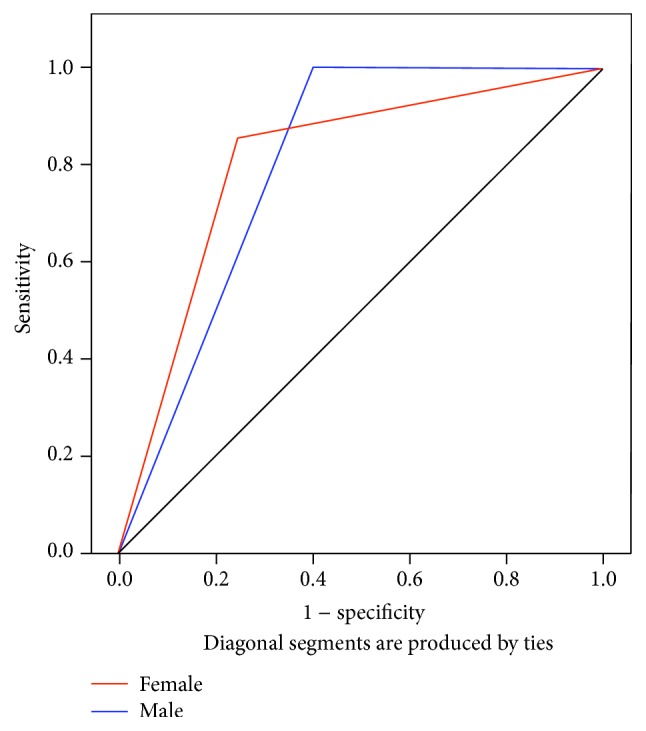
Receiver operating characteristic (ROC) curves of serum leptin and delayed puberty in *β*-TM patients with cut-off vales mentioned in the text. The red and blue lines indicate female and male *β*-TM patients, respectively. The AUCs (±SE) and *P* values for female and male *β*-TM patients are 0.717 (±0.166), *P* = 0.235 and 0.553 (±0.191), *P* = 0.777, respectively.

To assess the validity of low serum leptin levels as a predicator of short stature and delayed puberty in *β*-TM patients, cut-off values of 3.34 ng/mL and 4.52 ng/mL were selected for serum leptin in female patients aged ≤13 years and >13 years, respectively, whereas, for male *β*-TM patients, the threshold values for ≤13 years and >13 years were set at 2.45 and 3.09 ng/mL, respectively. Accordingly, the use of serum leptin to predict short stature showed sensitivity and specificity of 83.3% and 40% for male and 85.7% and 77.8% for female *β*-TM patients, whereas, for delayed puberty, sensitivity and specificity of 100% and 25% for male and 60% and 92.3% for female *β*-TM patients were observed. The receiver operating characteristic (ROC) curves for serum leptin in *β*-TM patients with short stature and delayed puberty are shown in Figures 3 and 4.

## 4. Discussion

Leptin plays a key role in controlling reproduction and the hypothalamic-pituitary-gonadal (HPG) axis, and the level of circulating leptin may affect the HPG axis [17]. These effects may highlight an essential role for leptin in regulating reproductive function [8, 9, 17], supporting the hypothesis that leptin is one of the factors mediating reproductive abnormalities in several disease states [18]. In addition, leptin is also part of a complex network of endocrine signals that includes several hormones and vitamin D, which governs the process of longitudinal bone growth [19].

The mean serum leptin level was found to be significantly lower in our *β*-TM patients than in control patients. Similar findings were reported for *β*-TM patients of different age groups [20–22]. A significantly lower level of serum leptin was also observed in our stunted *β*-TM patients. In addition, the low BMI values observed in our *β*-TM patients probably reflect a reduction in fat mass in these patients, resulting in low levels of leptin secretion. These associations may explain the importance of adipose tissue-dependent leptin secretion in normal growth.

Blüher and Mantzoros [18] found that, during early childhood, individuals who present a congenital leptin deficiency may demonstrate significant growth delay due to the decreased secretion of GH and decreases in the levels of IGF-1 and IGF-BP3. These results might suggest that leptin has both direct and indirect effects on the GH-IGF-I-insulin-like growth factor binding protein (IGF-BP) axis. Serum leptin has also been found to indirectly stimulate growth as a trigger for sexual development and, thus, the induction of the pubertal growth spurt [7].

The current study showed that *β*-TM patients with delayed puberty had significantly lower serum leptin levels than both *β*-TM patients with normal puberty and the control group. These findings are similar to those reported by Perrone et al. [20], who concluded that, in patients with *β*-TM, adipose tissue cannot ensure adequate leptin production when the highest leptin secretion levels are required during development. These authors suggested that the lack of leptin production might be a cofactor for the dysfunction in pubertal timing observed in patients with *β*-TM.

The present findings of significantly lower serum leptin levels in males with *β*-TM relative to females are similar to results observed in Greece [23] and Iran [24]. Serum leptin levels were found to be two- to threefold higher in pubertal girls relative to pubertal boys [25], which is also associated with a rise in oestrogen levels [26]. Androgens are thought to provoke a reduction in leptin production, which may explain the low levels of serum leptin observed in male individuals [27]. However, the difference in the serum leptin concentrations in both sexes also occurred during prepubertal age, implying that factors other than sex steroid hormones (e.g., fat mass in the body and energy expenditure) may cause a fluctuation in serum leptin concentrations [28, 29].

Linear multiple regression analysis showed a positive association between serum leptin levels and sex, BMI, and *β*-TM. Many studies [30, 31] showed that BMI is positively associated with high serum leptin levels. Gender also affects serum leptin levels [32] as leptin is higher in females than in males. The sex differences are probably due to different amounts of subcutaneous adipose tissue [33] and hormonal concentrations [26, 28]. However, *β*-TM appears to be an even stronger factor than sex as *β*-TM accounts for one-quarter (25.1%) of the variation in serum leptin levels. However, other reports mentioned that other factors can affect serum leptin levels, including lifestyle variables, serum lipid patterns, and iron overload [30, 34, 35]. Iron overload typically occurs in patients with *β*-TM due to repeated blood transfusions, which is accompanied by increased serum ferritin, a condition observed in our *β*-TM patients. Although iron is indispensable for life, it can act as a potent and potentially dangerous oxidant. Thus, iron overload followed by iron deposition in fat cells is harmful and toxic because of the free radical formation that causes the destruction of the adipocyte membrane and inhibits the activity of adipose cells [36]. The leptin receptor is also found on haematopoietic and bone marrow cells; hence, in *β*-TM patients, who display a high haemolytic rate due to an abnormal Hb structure, a defect in haematopoietic cells may result in decreased serum leptin levels [36]. Kyriakou and Skordis stated that the hypogonadotropic hypogonadism observed in *β*-TM is associated not only with the toxic effect of iron on gonadotroph cells but also with iron toxicity in adipocytes, thereby altering the physiological role of leptin in sexual maturation and fertility [37]. Dedoussis et al. found a negative correlation between soluble transferrin receptor levels, a clinical marker for total body iron stores, and plasma leptin in *β*-TM patients [38]. They reported that these findings enhance previous results indicating that leptin may play some role in haematopoiesis and could associate the pathophysiology of *β*-TM patients with the effect of leptin in triggering reproductive ability.

Recently, a direct relationship between iron and leptin was suggested [35]. This relationship is based on the observation that the absence of adipocyte ferroportin (iron-regulated transporter 1) results in increased levels of iron in the adipose tissue together with reduced serum leptin and the demonstration that the decreased leptin mRNA levels are due to decreased transcription, indicating that iron directly regulates leptin synthesis [35]. Whether such observation and results are applicable in *β*-TM patients requires further study.

Data from the HELENA study [39] of 967 healthy adolescents suggested that serum leptin levels could be used as a marker for the prediction of early-onset puberty. In our study, the sensitivity and specificity of serum leptin levels as a predicator of short stature and delayed puberty in *β*-TM patients were assessed using ROC analysis, which is considered a useful tool for evaluating the performance of diagnostic tests [40]. This analysis revealed higher sensitivity and specificity in *β*-TM females relative to males which was significant only in short-stature *β*-TM females. We are unable to explain the lower specificity of leptin levels in males with short stature or delayed puberty apart from differences in body fat distribution, particularly in the amount of subcutaneous fat, and sex hormones. The small sample size could also be a factor, particularly regarding delayed puberty. However, other factors cannot be excluded; thus, further investigation is required. Ganji et al. [41] suggested that physiological and/or metabolic differences could be the cause of variations in serum leptin levels between males and females.

Low serum leptin has implications for growth and pubertal development in normal children and adolescents [42]. These effects would likely be more pronounced in patients with *β*-TM. More data are required for an effective interpretation of our results. Furthermore, the interplay of leptin with the metabolism of energy balance and reproduction is well reviewed and discussed in several recent studies [8, 9, 17, 43] that investigated the mechanisms of leptin signalling through kisspeptin-dependent and kisspeptin-independent pathways and through leptin receptors. Whether these mechanisms are interrupted or amplified in thalassaemia patients remains an open question.

Our study has the following limitations. First, the sample size was small, which could affect our analysis and conclusions. Second, sex hormone profiles and measurements of fat distribution were not performed. Third, levels of the peptide hormone hepcidin, which is secreted by the liver and acts as a key regulator of iron metabolism in the body, as well as leptin receptor levels, were not determined. Despite these limitations, the results of our study are comparable to published findings and, to the best of our knowledge, our study is the first of its kind to be conducted in Arab patients with *β*-TM.

Importantly, every *β*-TM patient requires proper management and strict follow-up. Early diagnosis and treatment with chelating agents to prevent hypogonadism are essential for a better quality of life. A deeper knowledge of the pathogenesis and manifestation of *β*-TM, combined with innovative new treatments to prevent endocrine complications and iron overload toxicity, will result in better life for *β*-TM patients.

## 5. Conclusion

In conclusion, the association and interaction between serum leptin levels, growth retardation, and pubertal development in patients with *β*-TM suggested that serum leptin can be used to predict short stature in *β*-TM patients and as a guide for further therapeutic or hormonal modulation, particularly in females. Furthermore, additional research is required to elucidate the biochemical and physiological changes in subjects with *β*-TM that affect the levels of serum leptin.

## Figures and Tables

**Table 1:** Serum leptin, ferritin, age, weight, height, BMI *Z*-score, BMI, and IGF-1 in *β*-TM patients and controls according to sex.

Variables	Sex	*β*-TM patients	Controls	*P* value
	M/F	27/23	41/34	
Age (years)	Male	13.6 ± 3.4	13.7 ± 3.1	>0.05
	Female	12.3 ± 3.3	12.6 ± 3.0	>0.05

Height (cm)	Male	141.93 ± 15.54^b^	156.99 ± 16.07^b^	<0.001
	Female	132.11 ± 12.58	147.82 ± 14.45	<0.001

Weight (kg)	Male	34.81 ± 11.78	54.05 ± 19.55^b^	<0.001
	Female	30.52 ± 8.94	44.62 ± 15.96	<0.001

BMI *Z*-score	Male	−1.22 ± 1.15	0.42 ± 1.38	<0.001
	Female	−0.68 ± 1.04	0.09 ± 1.11	<0.004

BMI (kg/m^2^)	Male	16.79 ± 2.12	21.2 ± 4.65	<0.001
	Female	17.12 ± 2.62	19.85 ± 4.67	<0.014

S. leptin (ng/mL)	Male	1.71 ± 1.27^a^	3.92 ± 1.22^a^	<0.001
	Female	3.27 ± 1.97	5.95 ± 2.43	<0.001

S. ferritin (ng/mL)	Male	5285.5 ± 3220.2	114.14 ± 65.31^a^	<0.001
	Female	5084.0 ± 3260.5	67.74 ± 30.54	<0.001

The significant sex differences (males versus females) for the above parameters within each group of *β*-TM and controls were assessed as follows: ^a^ *P* ≤ 0.001, ^b^ *P* < 0.03.

**Table 2:** Serum leptin levels in patients with *β*-TM with delayed and normal puberty according to gender.

*β*-TM patients	Serum leptin levels (ng/mL)				*P* value
	Delayed puberty		Normal puberty		
	*N*	Mean ± SD	*N*	Mean ± SD	
Males (*N* = 11)	7	0.89 ± 0.66	4	1.68 ± 1.08	0.16
Females (*N* = 11)	6	2.32 ± 1.59	5	4.1 ± 1.88	0.12
All (*N* = 22)	13	1.55 ± 1.35	9	3.02 ± 1.96	0.032

**Table 3:** Serum leptin levels in *β*-TM patients with normal and short stature according to gender.

*β*-TM patients	Serum leptin levels (ng/mL)				*P* value
	Short stature		Normal stature		
	*N*	Mean ± SD	*N*	Mean ± SD	
Males (*N* = 27)	12	1.33 ± 1.07	15	2.0 ± 1.37	0.18
Females (*N* = 23)	14	2.32 ± 1.41	9	4.75 ± 1.83	0.002
All (*N* = 50)	26	1.87 ± 1.34	24	3.03 ± 2.04	0.021

**Table 4:** Stepwise multiple regression analysis between serum leptin levels and different variables in *β*-TM patients.

Variable	Beta	*R* ^2^	*P* value
Thalassaemia alone	0.512	0.256	<0.001
Thalassaemia + sex	0.399	0.413	<0.001
Thalassaemia + sex + BMI	0.289	0.478	<0.001
